# Priority setting: women’s health topics in multiple sclerosis

**DOI:** 10.3389/fneur.2024.1355817

**Published:** 2024-02-19

**Authors:** Lindsay Ross, Marcia Finlayson, Maria Pia Amato, Jeffrey Alan Cohen, Kerstin Hellwig, Mar Tintore, Sandra Vukusic, Amber Salter, Ruth Ann Marrie

**Affiliations:** ^1^Department of Neurology, Mellen Center for Multiple Sclerosis Treatment and Research, Neurological Institute, Cleveland Clinic, Cleveland, OH, United States; ^2^School of Rehabilitation Therapy, Queen’s University, Kingston, ON, Canada; ^3^Department of NEUROFARBA, Section of Neurosciences, University of Florence, Florence, Italy; ^4^IRCCS Fondazione Don Carol Gnocchi, Florence, Italy; ^5^Department of Neurology, Katholische Klinikum, Ruhr University, Bochum, Germany; ^6^Multiple Sclerosis Centre of Catalonia, Department of Neurology, Hospital Universitari Vall d’Hebron, Universitat Autonoma de Barcelona, Universitat de Vic-Universitat Central de Catalunya, Barcelona, Spain; ^7^Service de Neurologie, sclérose en plaques, pathologies de la myéline et neuro-inflammation-et Fondation Eugène Devic EDMUS pour la Sclérose en Plaques, Hôpital Neurologique Pierre Wertheimer, Hospices Civils de Lyon, Lyon, France; ^8^Centre des Neurosciences de Lyon, INSERM 1028 et CNRS UMR5292, Observatoire Français de la Sclérose en Plaque, Lyon, France; ^9^Université Claude Bernard Lyon 1, Lyon, France; ^10^Department of Neurology, Section on Statistical Planning and Analysis, UT Southwestern Medical Center, Dallas, TX, United States; ^11^Department of Medicine, Max Rady College of Medicine, Rady Faculty of Health Sciences, University of Manitoba, Winnipeg, MB, Canada; ^12^Department of Community Health Sciences, Max Rady College of Medicine, Rady Faculty of Health Sciences, University of Manitoba, Winnipeg, MB, Canada

**Keywords:** multiple sclerosis, women’s health, survey, focus groups, menopause, cancer

## Abstract

**Background:**

A scoping review found that most studies on women’s health in multiple sclerosis (MS) focused on pregnancy, fetal/neonatal outcomes and sexual dysfunction. Few studies addressed menopause, contraception, gynecologic cancers/cancer screening. However, the perceived relative importance of these knowledge gaps to people living with MS and other partners is unknown. We engaged a range of partners, including people living with MS, health care providers, researchers, and patient advocacy groups, to set priorities for future research in women’s health in MS.

**Methods:**

We employed a three-step global engagement process. First, we identified which broad research topics relevant to women’s health in MS were of highest priority using two surveys. Second, we developed specific research questions within these topics using focus groups. Finally, we prioritized the research questions with a third survey.

**Results:**

Overall, 5,266 individuals responded to the initial surveys [*n* = 1,430 global survey, mean (SD) age 50.0 (12.6), all continents; *n* = 3,836 North American Research Committee on Multiple Sclerosis survey, mean (SD) age 64.8 (9.6), United States]. Menopause, sexual dysfunction, pregnancy, gynecologic cancer/cancer screening, hormones and parenthood were identified as the most important topics. Focus groups generated 80 potential research questions related to these topics. In the final survey 712 individuals prioritized these questions. The highest priority questions in each research topic were: (i) How do perimenopause and menopause affect disease activity, course, response to disease-modifying treatment and quality of life in MS; (ii) What are the most effective strategies for managing issues around sexual intimacy, including related to low sexual desire, changes in physical function, and MS symptoms; (iii) Are there long-term effects of disease-modifying therapies on the children of persons with MS; (iv) What are the short and long-term effects of disease-modifying drugs on gynecologic cancer risk, particularly for high efficacy disease-modifying drugs and hematopoietic stem cell transplantation; (v) Are there hormone related treatments that can stabilize fluctuations in MS symptoms; and (vi) How does MS fatigue impact parenting strategies.

**Conclusion:**

Priorities for research relating to women’s health issues for persons with MS have been delineated using a collaborative process with key partners. Alignment of future research with these priorities should be monitored.

## 1 Introduction

Multiple sclerosis (MS) affects over 2.8 million persons worldwide (1), of whom an estimated three-quarters are women. These women will need to manage the stages of their reproductive life, including pregnancy and menopause, as they cope with MS. In our prior scoping review of the literature regarding topics relevant to women’s health in MS (2), most studies focused on pregnancy, fetal/neonatal outcomes and sexual dysfunction (3). Few studies addressed relevant topics such as menopause, contraception, gynecologic cancers and cancer screening. The generalizability of prior studies was limited with respect to geography, race and ethnicity, and progressive MS.

The perceived relative importance of these knowledge gaps to people living with MS and other stakeholders is unknown. Engagement of people with MS and clinicians in priority setting for research has the potential to improve the relevance, quality, uptake and impact of the research conducted (4, 5). Several validated approaches exist for collaborative priority setting involving various stakeholders, including patients and researchers (4) including the James Lind Alliance Priority Setting Partnership (JLA). The JLA approach is highly utilized and identifies current gaps in knowledge using surveys and then brings partners together for workshops to create and prioritize research questions in response (5).

We aimed to engage a range of partners, including people living with MS, health care providers, researchers, and patient advocacy groups, to set priorities for future research in women’s health in MS, building on our prior scoping review. We adapted our approach from the model used by the JLA to generate and prioritize research questions. Specifically, we used virtual focus groups and a follow-up survey rather than a single workshop to optimize global engagement.

## 2 Methods

### 2.1 Design

We employed a three-step process to engage stakeholders globally (Supplementary Figure S1). First, a steering committee made up of members of the International Advisory Committee on Clinical Trials in MS, and external members with expertise in qualitative methods and survey design was formed to guide the work. The committee sought to identify which broad research topics relevant to women’s health in MS were of highest priority using two cross-sectional surveys. Second, we developed specific research questions within these priority topics using focus groups. Finally, we prioritized the research questions using a third cross-sectional survey. These steps are delineated further below.

### 2.2 Setting & populations

First, we distributed an anonymous online questionnaire to various stakeholders including individuals with MS, care partners for people with MS, clinicians, researchers, and patient advocacy organizations; we aimed to distribute the questionnaire globally (hereinafter the “global” survey). We asked multiple organizations to distribute the link to the questionnaire via their usual communication channels. This included the National Multiple Sclerosis Society, Consortium of MS Centers, Americans Committee for Treatment and Research in MS, European Committee on Treatment and Research in MS, Latin American Committee on Treatment and Research in MS, Pan-Asian Committee on Treatment and Research in MS, Multiple Sclerosis International Federation (MSIF), the MS Society of Canada, International Women in MS, Societe Francophone de la Sclerose en Plaques and ARSEP Foundation. The questionnaire was also distributed to neurologists in Africa, and the German Neurological Society. The survey included a statement indicating that completion of the survey implied consent.

Second, we included a questionnaire in the Spring 2022 update survey distributed to participants in the North American Research Committee on Multiple Sclerosis (NARCOMS) Registry. The NARCOMS Registry is a self-report registry for persons with MS (6). Participants agree that their de-identified information can be used for research. NARCOMS participants complete questionnaires at enrollment and update their demographic and clinical information via semi-annual surveys. These questionnaires are completed on paper or online as desired by the participant.

The Institutional Review Board at UT Southwestern approved the global survey and the NARCOMS survey.

### 2.3 Survey

The global survey assessed respondent characteristics including gender, age, and country of resident for all participants (Appendix I). Respondents reported whether they had MS, were a caregiver for someone with MS or neither. Respondents with MS reported their type of MS (clinically isolated syndrome, relapsing remitting, secondary progressive, primary progressive and do not know) using a question used by the NARCOMS Registry. Respondents without MS who were not caregivers were asked what sector they worked in (government, health care, non-profit organization, research in academic or health care setting, research in commercial/industry setting, industry setting non-research, and other). They also reported their professional background and organizations with which they were affiliated.

The research topics considered included puberty, menstrual cycle, contraception/birth control, pregnancy (including pregnancy loss), neonatal outcomes/childhood development, fertility/infertility, assisted reproduction, menopause and hormone replacement therapy, sex hormones and influence on MS outcomes or as a treatment, sexual dysfunction, parenthood, family planning services, gynecologic cancer and cancer screening, sexually transmitted diseases, sexual orientation, gender identity, and intimate partner violence. These research topics were selected based on the prior scoping review (2). To establish research priorities, the global survey asked the following: “With respect to the issue of women’s health in MS, what are the *most important* research areas in general? For example, if you thought one of the most important research questions was whether menopause affects symptoms of MS you would rank menopause as important. Please rank your top 5 choices in order of importance (#1 being the most important).” Respondents were also invited to write-in other topics that were not listed in the ranking question. Finally, we asked respondents to indicate if they were interested in participating in subsequent initiatives related to women’s health in MS. Interested respondents provided their contact information using a separate data collection form. The survey took approximately 5 minutes to complete, and was brief to enhance completion.

We included the same research priority ranking question from the global survey in the Spring 2022 NARCOMS survey, specifically for women participants. Since participants in the NARCOMS Registry typically complete longer questionnaires, they offered the opportunity to better understand the factors associated with responses to the ranking question. We obtained information regarding gender, age, region of residence, race and ethnicity, education level and age of symptom onset from the NARCOMS enrollment survey. We obtained information about annual household income, comorbidities, health behaviors, and disability status based on Patient Determined Disease Steps (7, 8) from the Spring 2022 questionnaire (Appendix II).

### 2.4 Survey administration

The surveys were developed and managed using REDCap (Research Electronic Data Capture) hosted at the University of Texas Southwestern. REDCap is a secure, web-based software platform developed to support data capture for research studies (9). The global survey was pilot tested by 10 individuals who were not involved in developing the survey. Following the pilot test, minor adjustments were made to formatting, and wording of topics listed in the ranking question. Specifically, font size was enlarged in some headers, some punctuation was corrected, care partner was changed to caregiver, the question “Are there other general topics that should be considered that …?” was changed to “Are there other general topics on women’s health that…,” and headings for rankings were modified to include a number and rank (not just a number) as follows: 1 (Top Priority). The global survey was distributed from March 2022 through June 2022, initially in English. After forward translations were obtained from a native speaker, they were back translated by a different translator to ensure accuracy, and the survey was subsequently distributed in French and Spanish via the MSIF, Société Francophone de la Sclérose en Plaques and ARSEP Foundation. The NARCOMS survey was distributed from April 2022 through July 2022 in English.

### 2.5 Focus groups

Following the identification of the priority research topics via the global and NARCOMS surveys, we held focus groups to develop specific research questions within those topics. Ultimately, we included six topics rather than five, due to similarity of rankings for two of the topics. We used two strategies. First, members of the Multiple Sclerosis International Federation’s International Medical and Scientific Board (MSIF IMSB) were asked to suggest relevant questions for each topic. These questions were collated and discussed in parallel focus groups; each group was facilitated by a member of the IMSB or a member of the steering committee (RM). Second, at large focus group participants were drawn from the pool of 367 individuals who had responded to the global survey indicating interest in future initiatives. All of these individuals were invited via email to participate with a REDCap link to signup; participation implied consent and this was detailed in the invitation email (distributed in English, French and Spanish after translation by a translator from an accredited organization, KeyLingo) and reviewed at the start of the focus group.

The characteristics of the focus group participants drawn from the global survey respondents were captured using a survey administered via REDCap before the focus group with confirmation of completion confirmed at beginning of the focus group (Appendix III); characteristics of the IMSB focus group participants were not captured. The characteristics captured included gender, race, ethnicity, age, country of residence, stakeholder group, and, if relevant, their current MS disease course. We aimed to hold five to six focus groups, each involving 3–10 participants. Groups were held on a password-protected virtual platform. We opted for virtual rather than in-person meetings to enhance convenience for participants, to limit costs, and to allow people to participate from geographically-diverse areas. Focus groups were led by an experienced facilitator (MF) with a semi-structured interview guide.

Research questions generated by each group were compiled to create a master list of questions. Similar questions were synthesized into a single question by members of the study steering committee with agreement by two members.

The Institutional Review Board at the Cleveland Clinic approved the focus groups including the corresponding demographic survey.

### 2.6 Final survey

Using the same approach as for the initial global survey, we distributed a third survey that asked respondents to rank order the specific research questions, as generated by the two sets of focus groups, within each research topic. Respondents also reported their characteristics as they had in the initial survey except with expansions of options for gender and ancestry consistent with the focus group survey. The survey was distributed in English, French and Spanish to all organizations that shared the invitation on our behalf in August and September 2023.

### 2.7 Analysis

For all surveys, we used descriptive statistics to summarize responses, including mean [standard deviation (SD)], median [interquartile range (IQR) and frequency (percent)]. Bivariate analyses used student’s *t*-tests, Wilcoxon or Kruskal–Wallis tests and chi-square tests, as appropriate.

To examine patterns of responses from the initial global survey according to respondent characteristics we used multivariate analysis of covariance (MANCOVA) with Pillai’s trace as the test statistic, which reduced the number of hypothesis tests. Because the outcome variables were ordinal, we rank ordered the responses before including them in the model. Missing values, which represented a ranking that was not in the top five were assigned a value of 6 before the ranking was conducted. Independent variables for the global survey were age (continuous), gender and type of stakeholder (person with MS, caregiver, other). For comparability to the NARCOMS survey we repeated the analysis of the global survey limited to respondents with MS. Independent variables for the NARCOMS survey were age (continuous), education, income, race, alcohol intake, physical activity, smoking status, disease modifying therapy (DMT) use, number of physical comorbidities, depression, anxiety, and disability status, as defined above. We assessed model assumptions (multivariate normality, linearity of relationships between dependent variables, and homogeneity of variance and covariance) using standard methods.

For the final global survey, if a respondent failed to rank all questions in a category, we assigned the lowest possible ranking to the missing questions (thus creating ties for those questions), before generating the rankings across all respondents.

Statistical analyses were performed using SAS V9.4 (SAS Institute Inc., Cary, NC) and R 4.0.3 (R Core Team 2023).

## 3 Results

### 3.1 Respondents of the global survey

**Figure 1:**
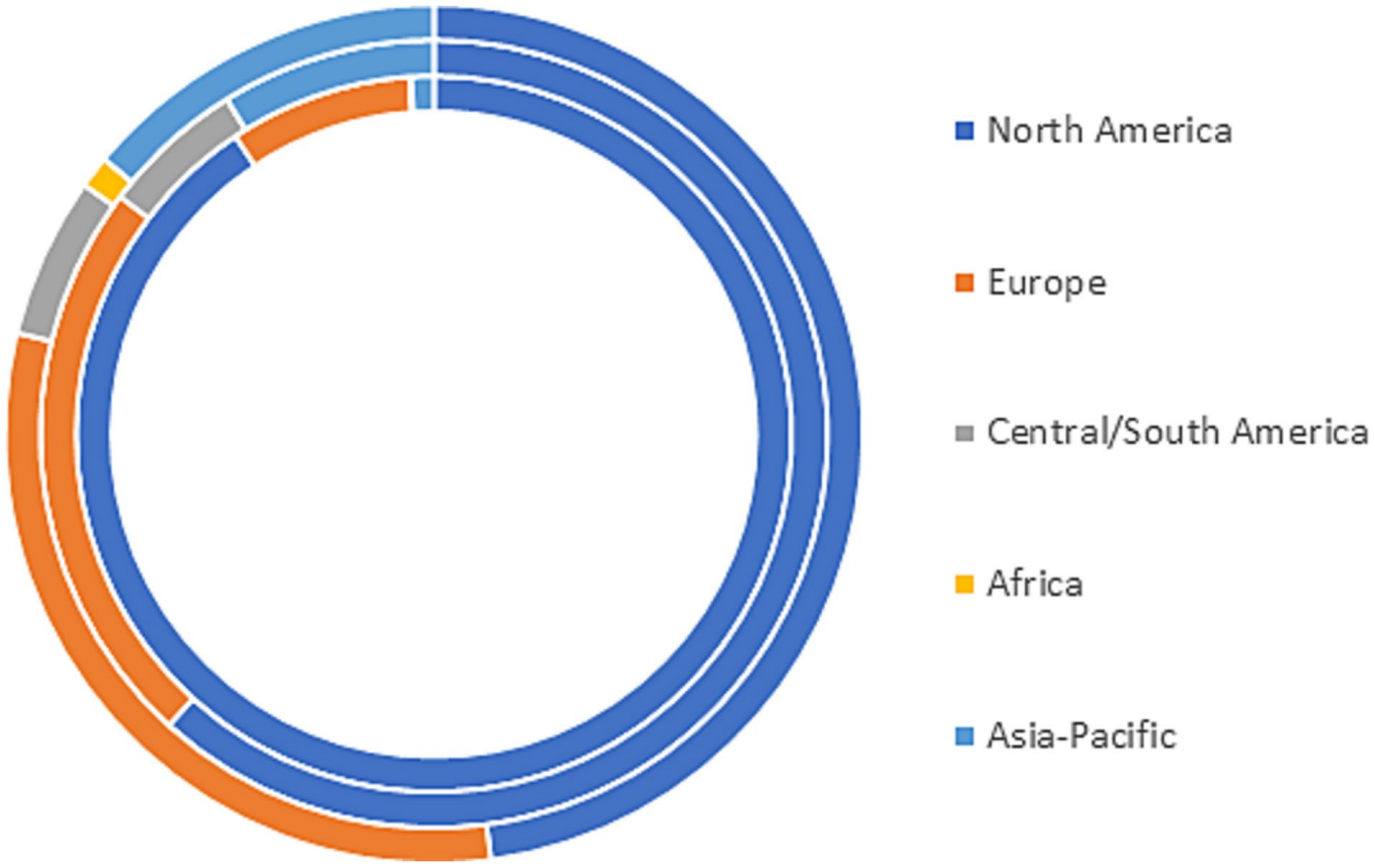
Geographic distribution of respondents to first global survey. Inner ring = persons with multiple sclerosis, middle ring = care partners, outer ring = all others.

Overall, 1,430 individuals responded to the global survey (Table 1). Most respondents identified as female, and were living with MS. The most common geographic region represented was North America (84.7%), followed by Europe (11.5%), and Asia-Pacific (2.7%) regions. We observed differences in geographic region represented according to the type of respondent (Figure 1). Ninety percent of respondents with MS were from North America, and 8.3% were from Europe. Among care partners, 61.8% were from North America, and 23.5% were from Europe. Among the 1,206 respondents with MS, 878 (73.3%) had relapsing remitting MS. Among the 174 respondents who did not have MS and were not care partners for someone with MS, nearly 75% were health care providers, predominantly physicians. Over 40% conducted research in academic, health care or commercial settings.

**Table 1:** Characteristics of respondents of the global survey.

Characteristic	All *N* = 1,430	Person with MS *N* = 1,206	Care partner *N* = 35	Other *N* = 174
Age (years)^*^, mean (SD)	50.0 (12.6)	50.0 (12.6)	51.7 (12.4)	48.8 (11.6)
**Gender** ^**a**^ **, *n* (%)**				
Female	1,353 (95.1)	1,191 (98.9)	21 (60.0)	130 (75.1)
Male	69 (4.8)	12 (1.0)	14 (40.0)	43 (24.9)
Neither male nor female	1 (0.07)	1 (0.1)	0 (0)	0 (0)
**Type of respondent** ^**b**^ **, *n* (%)**				
Person with MS	1,206 (85.2)			
Care partner	35 (2.5)			
Not a person with MS or care partner	174 (12.3)			
**Type of MS** ^**c**^ **, *n* (%)**				
Clinically isolated syndrome		27 (2.3)		
Relapsing remitting		878 (73.3)		
Secondary progressive		172 (14.4)		
Primary progressive		84 (7.0)		
Do not know/unsure		37 (3.1)		
**Work sector** ^**d**^ **, *n* (%)**				
Government				4 (2.3)
Health care/health professional				130 (74.7)
Non-profit				16 (9.2)
Research in academic/health care setting				74 (42.5)
Research in commercial/industry setting				3 (1.7)
Industry setting, non-research				1 (0.6)
Other				1 (0.6)
**Organizational role** ^**d**^ **, *n* (%)**				
Administrative				11 (6.3)
Clinical care				134 (77.0)
Patient advocacy				9 (5.2)
Research and evaluation				100 (57.5)
Other				6 (3.5)
**Professional background** ^**d**^ **, *n* (%)**				
Administrator				4 (2.3)
Physician				121 (69.5)
Nurse				18 (10.3)
Physiotherapist				1 (0.6)
Occupational therapist				1 (0.6)
Social worker				0 (0)
Speech therapist				1 (0.6)
Researcher—clinical				60 (34.5)
Research—health systems and services				10 (5.8)
Researcher—biomedical	25 (14.4)			
Researcher—population health/EPI	21 (12.1)			
Other	9 (5.2)			
**Affiliation**				
TRIMS^**^	144 (10.1)			
AAN	89 (6.2)			
Chinese Neurol. Assoc.	2 (0.1)			
CMSC	89 (6.2)			
EAN	33 (2.3)			
ECF	22 (1.5)			
EFNS	6 (0.4)			
EMSP	21 (1.5)			
GNS	4 (0.3)			
ITRIMS	3 (0.2)			
iWiMS	61 (4.3)			
MSIF	46 (3.2)			
MS societies	699 (48.9)			
RIMS	11 (0.8)			
Other	89 (6.2)			
None	517 (36.2)			

^*^One implausible value removed; ^a^7 missing; ^b^15 missing; ^c^8 missing; ^d^multiple responses possible. ^**^TRIMS include Americas Committee for Treatment and Research in MS (ACTRIMS), Brazilian Committee for Treatment and Research in MS (BCTRIMS), European Committee for Treatment and Research in MS (ECTRIMS), Latin American Committee for Treatment and Research in MS (LACTRIMS), Middle East North Africa Committee for treatment and Research in MS (MENACTRIMS), Committee for treatment and Research in MS MEXTRIMS, Pan-Asian Committee for treatment and Research in MS (PACTRIMS), Russian Committee for treatment and Research in MS (RUCTRIMS), Sri Lanka Committee for treatment and Research in MS (SLTRIMS). Note some individuals reported these affiliations even if the survey was not directly distributed to those organizations. AAN, American Academy of Neurology; CMSC, Consortium of MS Centers; EAN, European Academy of Neurology; ECF, European Charcot Foundation; GNS, German Neurological Society; iWiMS, International Women in Multiple Sclerosis; MS = multiple sclerosis; RIMS, Rehabilitation in MS; EFNS, European Federation of Neurological Societies; EMSP, European Multiple Sclerosis Platform.

### 3.2 Participants in NARCOMS survey

Of 8,597 persons to whom the NARCOMS survey was distributed, 5,098 (59.3%) responded. As compared to respondents, non-respondents were 2.9 years younger on average, less likely to identify as White, to have a lower level of education, and to have more severe disability (Supplementary Table S1). Of the 5,098 participants, 3,836 were females diagnosed with MS who had provided complete information regarding age and gender. Most female participants were aged 65 years and older, self-identified as White, with a post-secondary education and moderate or severe disability (Table 2).

**Table 2:** Female participants in the NARCOMS survey.

Characteristics	Females
*N*	3,836
Age at time of spring 2021 survey (years), mean (SD)	64.8 (9.6)
**Age group (years), *n* (%)**	
18–34	50 (1.3)
35–49	253 (6.6)
50–64	840 (21.9)
≥65	2,693 (70.2)
Female, *n* (%)	—
**Race, *n* (%)**	
White	3,352 (87.4)
Black	91 (2.4)
Other	393 (10.2)
**Education at enrollment, *n* (%)**	
High school/GED	987 (26.7)
Post-secondary	2,707 (73.3)
**Annual household income at enrollment, *n* (%)**	
Less than $50,000	1,163 (30.3)
≥$50,000	1,633 (42.6)
I do not wish to answer	1,040 (27.1)
**Region, *n* (%)**	
United States	3,782 (98.6)
Outside United States	54 (1.4)
Age at MS Symptom onset (years), mean (SD)	31.0 (9.8)
Age at MS diagnosis (years), mean (SD)	38.5 (9.7)
Disease duration (years), mean (SD)	33.8 (11.4)
PDDS, median (IQR)	3 (1–6)
**PDDS, *n* (%)**	
Mild (0–1)	1,169 (31.2)
Moderate (2–4)	1,192 (31.8)
Severe (5–8)	1,383 (36.9)
Any disease-modifying therapy in last 6 months, n (%)	1919 (50.0)
Current smoker, *n* (%)	212 (5.6)
Any leisure activity, *n* (%)	2091 (55.4)
**Alcohol intake, *n* (%)**	
Never	1,473 (39.0)
Up to 2–4 times a month	1,530 (40.5)
≥2 times a week	779 (20.6)
**No. physical comorbidities, *n* (%)**	
0	853 (22.2)
1	1,185 (30.9)
2	884 (23.1)
≥3	914 (23.8)
Anxiety, *n* (%)	819 (22.0)
Depression, *n* (%)	1,417 (38.1)

GED, General Educational Development; PDDS = Patient Determined Disease Steps.

### 3.3 Research topic ranking

**Figure 2:**
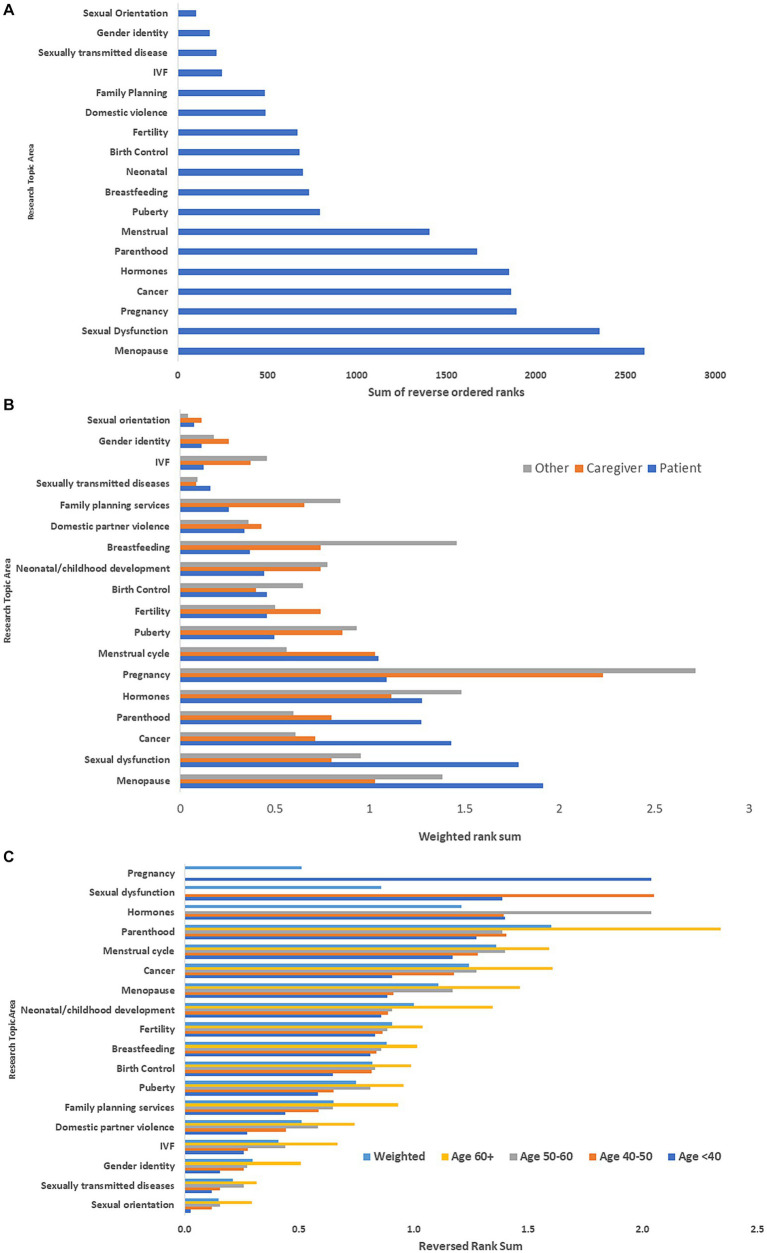
**(A)** Rank ordering of research priorities: global survey^*^. **(B)** Rank ordering of research priorities stratified by type of respondent: global survey^**^. **(C)** Rank ordering of research priorities stratified by age of respondent with multiple sclerosis: global survey^***^. ^*^Ranks were reversed ordered then summed. Highest sum indicated highest priority topic. ^**^Ranks were reversed ordered then summed. These were weighted by number of participants to place responses from each group on the same scale. Highest sum indicated highest priority topic. ^***^Ranks were reversed ordered then summed. These were weighted by number of participants in each age group. Highest sum indicated highest priority topic. IVF, *in vitro* fertilization.

Among respondents to the global survey, the top five research topics were menopause, sexual dysfunction, pregnancy, gynecologic cancer/cancer screening, and hormones (Figure 2A). Differences in priority ranking by type of respondent are illustrated in Figure 2B. Respondents with MS identified menopause, sexual dysfunction, pregnancy, gynecologic cancer/cancer screening, and parenthood as the top five topics. Caregivers identified pregnancy, hormones, menstrual cycle, menopause and puberty as the top five topics. Clinicians and researchers identified pregnancy, hormones, breastfeeding, menopause, and sexual dysfunction as the top priorities.

Among the respondents with MS, research priorities differed by age group (Figure 2C). The highest priorities among those <40 years old were pregnancy, sexual dysfunction, parenthood, sex hormones and the menstrual cycle, whereas they were parenthood, menstrual cycle, menopause, cancer and child development for those aged ≥60 years. However, when weighting topic prioritization by age three of the five highest priority topic areas remained the same as compared with the overall non-weighted rankings-gynecological cancer/cancer screening, menopause, and hormones.

On multivariate analysis, respondent type, and age of the respondent remained associated with different priority rankings overall (Table 3). Gender and region were also associated with different patterns of priority rankings. When we examined the association of individual factors with ranking of specific research topics, age and respondent type were associated with the ranking of most topics (Supplementary Table S2). Gender was associated only with rankings for menopause, pregnancy, breastfeeding, domestic violence, gender identity and sexual orientation; females ranked menopause higher than males, but ranked all other topics lower than males did. Region was only associated with rankings for sexual dysfunction, sex hormones, and neonatal outcomes; sexual dysfunction and sex hormones were ranked higher by participants from North American compared to other regions. When we limited the analysis to female respondents with MS, the findings were similar (Supplementary Table S3).

**Table 3:** Factors associated with priority rankings in MANCOVA analysis: overall effect^*^.

Factor	Global survey *F*-value (df), *p*-value	Global survey^**^ *F*-value (df), *p*-value	NARCOMS survey^**^ *F*-value (df), *p*-value
Type of participant	**6.57 (36), <0.0001**	—	—
Region	**2.25 (18), 0.002**	**2.60 (18), 0.0003**	
Gender	**2.18 (36), <0.0001**	—	—
Race	—		0.80 (36), 0.80
Age	17.3 (18), <0.0001	19.3 (18), <0.0001	**12.2 (18), <0.0001**
Education	—		**2.50 (18), 0.0004**
Income	—		**2.92 (36), <0.0001**
No. physical comorbidities	—		1.09 (54), 0.30
Anxiety	—		1.06 (18), 0.39
Depression	—		**2.95 (18), <0.0001**
Physical activity	—		**2.95 (18), <0.0001**
Smoking status	—		1.33 (18), 0.16
Alcohol intake	—		**1.46 (36), 0.036**
Disability status	—		**1.43 (36), 0.048**
Disease-modifying therapy use	—		**2.30 (18), 0.0014**

^*^MANCOVA provides evidence of overall effect, but not directionality. ^**^Sample restricted to women with MS. Bold indicates statistical significance.

Overall, participants in the NARCOMS survey (all females with MS), identified the same top five research topics as the global survey. The multivariate analysis of the NARCOMS survey similarly showed that age influenced the research topics that were prioritized (Table 3). Also, education, income, depression, disability status, disease-modifying therapy use, physical activity and alcohol intake were associated with the topics prioritized.

### 3.4 Focus groups

Of the 367 individuals who had previously indicated interest in related research following the global survey 87 (24%) responded indicating interest in the focus groups with 82 persons (94%) selecting English language preference. Among these, 54 individuals (62%) completed the follow-up survey collecting demographic information and availability for focus group sessions.

**Figure 3:**
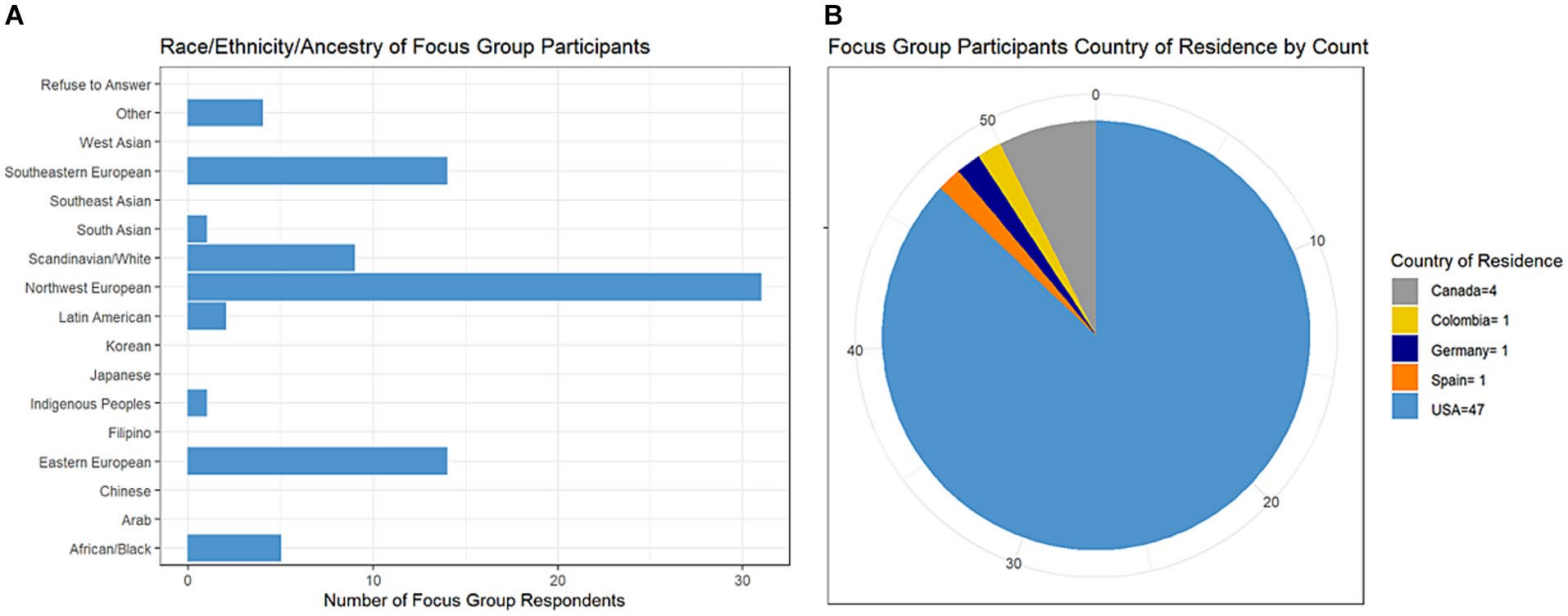
Characteristics of focus group survey respondents.

All respondents were women with average age 50 years (range 31–74). By stakeholder type, 51 individuals indicated being a person with MS, 2 were care partners for a person with MS, 5 were clinicians, 2 were patient advocates, and 1 was a researcher (responses were not mutually exclusive). Most persons with MS indicated having relapsing-remitting course (*n* = 41/51, 84%). Many respondents reported European or Scandinavian backgrounds followed by African/Black background with limited or no representation from other backgrounds (Figure 3A). Nearly all respondents were from the USA (Figure 3B); individuals from the USA were from 23 different states with California having the highest number of respondents at 8.

Participants were selected based on availability, and ultimately five focus groups were held all in English due to insufficient numbers of participants to compose groups in other languages. A total of 38 individuals were invited across the five sessions with 19 persons arriving and participating in the sessions. The focus groups generated between 21–37 research questions each covering at least five of the six high priority topics.

The MSIF IMSB’s focus groups generated 101 research questions.

All research questions were collated and summarized by the steering committee into a final list of 80 research questions split across the six topics.

### 3.5 Respondents of the final survey

Overall, 712 individuals responded to the second global survey (Supplementary Table S4). Most respondents identified as women, and two-thirds were living with MS. The most common geographic region represented was Europe (51.9%), followed by North America (33.2%), and Central/South America (9.2%). We observed differences in geographic region represented according to the type of respondent (Supplementary Figure S2), with the highest proportion of people with MS being from Europe, whereas the highest proportion of care partners and other respondents were from North America. Among the 233 participants who did not have MS and were not care partners for someone with MS, over 70% were health care providers.

### 3.6 Research question ranking

Table 4 shows the questions that were developed from the focus groups, ordered by their priority after the second global survey.

**Table 4:** Rank ordering of research questions developed by focus groups, organized according to topic.

Topic area	Research questions
Menopause	How do perimenopause and menopause affect disease activity, course, response to disease-modifying treatment and quality of life in MS? How does MS affect the experience of menopause (e.g., symptoms, severity)? Do MS disease-modifying therapies or symptomatic therapies alter ovarian function, or the symptoms or timing of menopause? Are there potential treatments for menopause symptoms that can also help with MS symptoms (e.g., oxybutynin for night sweats and bladder dysfunction)? What is the impact of hormone replacement therapy on disease activity, course, and progression in MS? How effective and how safe are hormonal therapies for menopause in women with MS? What is the symptom overlap between menopause and MS, and is the management different? How can people with MS prepare for/ plan for menopause? What, if any, is the difference in induced menopause vs. natural menopause on MS disease activity? What is the impact of heat intolerance on the experience of hot flashes? What is the role, safety and effectiveness of complementary approaches/supplements to address menopause/menopausal symptoms?
Sex hormones	Are there hormone related/hormone altering treatments that can stabilize fluctuations in MS symptoms? What is the optimal contraceptive strategy for women with MS? What impact do medications used to manage MS have on hormones? What is the role of estrogen, progesterone and other hormones in the risk of MS, risk of disease activity, progression and fatigue? How can education and resources be provided to women with MS surrounding impacts of hormone changes in MS? Do hormone levels affect response to disease-modifying therapy? How could diet or vitamin D affect the menstrual cycle, and how might that in turn affect MS? How can we differentiate MS symptoms and symptoms of hormone abnormalities? How does puberty affect MS risk, or risk of increased disease activity? Are there differences in menstrual symptoms for women with MS? Does menstruation worsen MS, cause pseudorelapses, or cause symptom worsening? Is pre-menstrual dysphoric disorder (PMDD) more common or worse in women with MS, and does this differ by age group? What is known about progesterone hypersensitivity in people with MS? Does the gender of the physician affect how hormone issues in MS are handled? Is there an association between severity of menstrual symptoms and subsequent development of MS? Do transgender individuals who take sex hormones to align their body with their gender alter their risk of developing MS or their course of MS?
Sexual dysfunction	What are the most effective strategies for managing issues around sexual intimacy, including related to low sexual desire, changes in physical function, and MS symptoms? What role do pelvic pain, pelvic floor spasticity, and pelvic floor physiotherapy play in sexual dysfunction in MS? How common is sexual dysfunction in MS and what are the risk factors including changes on MRI? What are the information and knowledge needs of providers to support sexual intimacy for persons with MS, and when in the disease is the best time to have discussions around sexual dysfunction? Are there effective preventative strategies for the development of urinary leakage or sexual dysfunction for women with MS? How can women with MS be supported in partner communication related to sexual dysfunction at different stages in an intimate relationship? What is the best way to assess sexual dysfunction in MS? How can sexual dysfunction from MS versus sexual dysfunction from medications used to treat MS, MS symptoms and menopause be disentangled? Why do MS symptoms change during sexual activity, how much of it is due to heat? What is the best way to give partners of women with MS information and support around sexual dysfunction in MS? How can we normalize discussion around women? How often do women with MS and health care providers talk about sexual dysfunction? How often are women with MS referred to sex therapists and how can sex therapists be integrated in MS care?
Pregnancy	Are there long-term effects of disease-modifying therapies on the children of persons with MS? How do MS and disease-modifying therapies affect the ability to become pregnant, the course of the pregnancy, labor and lactation? What factors do women with MS consider when making decisions surrounding pregnancy and how can they be supported in making those decisions? What is the best treatment strategy in women with highly active disease planning a pregnancy? Which disease-modifying and symptomatic therapies are safe during pregnancy and breastfeeding? What underlying disease process or treatment factors influence the effects of pregnancy and breastfeeding on MS, and the risk of post-partum relapse? How can pregnancy/ post-partum fatigue be managed for persons with MS? How does birth control affect people with MS? How does pregnancy affect symptoms of MS? Are women with MS more likely to need IVF/assistive reproductive technologies, how does IVF impact persons with MS, and what information about IVF can be provided to support decision-making? What is the optimal time period to breastfeed post-partum before starting a disease-modifying therapy? What is the knowledge base globally among obstetricians/mid-wives of fetal risk of disease-modifying therapies and common symptomatic medications used for MS? How does the experience of pregnancy in women with MS compare to that of women without MS? What is the interplay between stopping birth control in conjunction with stopping disease-modifying therapy for pregnancy? Does pregnancy affect the rate of being diagnosed with MS in people with radiologically isolated syndrome or clinically isolated syndrome (first attack)? Are there diagnostic delays for pregnant/post-partum individuals presenting with MS? What are the challenges in positioning for pregnancy visits and the birth and how can these be adapted?
Parenthood	How does MS fatigue impact parenting strategies? How does the health of a parent with MS affect their children? What communication strategies or programs can be designed for children or grandchildren of persons of MS to help them understand the disease and provide support to these individuals? What information and support do people with MS need to be supported in decision-making surrounding becoming parents, preparing to manage parenthood and planning ahead for the future? How can we predict the risk of a child of someone with MS, and how can we reduce the risk? How does MS in general affect parenting (e.g., teaching kids skills on a different timeline)? What are the system level resources needed for persons with MS to support parenthood, including single parenthood? What are strategies that could be effectively used to increase confidence/safety of caring for children/grandchildren/nieces/nephews for persons with MS (strategies to address fatigue, balance, mobility, strength and other symptoms)? What are approaches that can help women with MS experiencing guilt, shame, or grief around issues related to motherly roles? What differences are there in concerns/worries about parenting and children for persons with MS? What are effective communication strategies in discussing parenthood concerns for persons with MS with their provider and with their family? In what ways to do the current parenting culture align/misalign with caring for yourself as women with MS?
Gynecological cancer and screening	What are the short and long-term effects of disease-modifying drugs on gynecologic cancer risk, particularly for high efficacy disease-modifying drugs and hematopoietic stem cell therapy? What disease-modifying therapies should be used in patients with active gynecologic cancer? How often should cancer screening be done for women using disease-modifying therapies and how long should this continue after stopping disease-modifying therapies? What access barriers are there for persons with MS to getting cancer screenings and treatment? How healthcare providers be trained to support individuals with physical disabilities as they access cancer care, and how can accessible exam tables be designed for people with MS? Are the risks and symptoms of gynecologic cancers different for women with and without MS? Is the human papillomavirus (HPV) vaccine well tolerated by women with multiple sclerosis and should they receive Gardasil vaccination against HPV? How can cancer treatments and the treatment environment be adapted to accommodate for MS symptoms including additive symptoms from cancer/treatment? What are the experiences of persons with MS undergoing gynecologic cancer, screening, and treatment? How can we educate persons with MS and their caregivers about support resources, advocacy strategies and leave options when they are dealing with cancer, screening, treatment? What are the needs/challenges of caregivers of persons with MS undergoing cancer, screening, and treatment?

## 4 Discussion

We conducted a mixed method, multi-partner effort to identify priority research questions focused on women’s health issues for persons with MS (pwMS). In two large surveys with over 5,000 total respondents, we identified that women’s health issues of menopause, sexual dysfunction, pregnancy, gynecologic cancer/cancer screening, hormones and parenthood were the priority topics of interest for further research. Moreover, focus groups and a follow-up survey prioritized questions for each topic area. Some top questions focused on how MS may affect the symptoms and/or severity of menopause, impacts of fatigue on parenting strategies, interest in the possibility of hormone-related treatments ability to stabilize MS symptom fluctuations and others. We urge funders and researchers to direct future efforts to answer these newly identified high priority research questions.

Notably, we identified discordance in the priorities of different stakeholder groups. All stakeholder groups agreed that menopause and pregnancy were high priority topics for further study. However, gynecological cancer and screening was only a top priority for pwMS while sex hormones were only a top priority for clinicians/researchers and care partners. Differences in priorities by stakeholder groups have been noted in the past in other areas (10) and was among the factors prompting the development of the JLA. Pertaining to MS specifically, patients and physicians are known to have value judgment differences when it comes to body functions (11), treating MS symptoms (12), and evaluating burdens of disease-modifying therapies (13). Also, pwMS and their care partners have reported differences in how unmet needs should be addressed (13, 14). While we did not specifically investigate underlying reasoning behind stakeholder prioritizations in this study, it is likely these different valuations and experiences surrounding the disease played a role. Similarly, valuation differences were noted by age, gender, and region of the world of respondents. These results highlight the importance of engaging a diverse set of stakeholders and the value of formal prioritization work.

The top research area priorities of pwMS, particularly menopause and gynecological cancer, reflect current knowledge gaps (2). Of 353 studies included in a scoping review of women’s health in MS over the period 1983 to 2020, only 10 were related to menopause and 8 to gynecologic cancer/cancer screening. Similar misalignments between the priorities of pwMS and existing research have been noted in other MS topics. For instance, with respect to wellness pwMS were noted to be strongly interested in information on diet and cannabis use but substantial gaps in knowledge existed in these areas (15). In women’s health generally patient priorities have also aligned with areas of relative gaps in the topics of endometrial cancer (16), miscarriage (17), and stillbirth (18). Now that the priorities of pwMS are known it will be important to monitor whether future research aligns with the final priority list.

Menopause emerged as a consistent priority area across all stakeholder types and in the age –weighted evaluation. Given the typical onset of MS in young adulthood and that pwMS undergo menopause at the same age as controls (19), most women will be diagnosed before menopause. Therefore, menopause may be the most common women’s health issue experienced for pwMS. Menopause may also influence disease course; some studies have reported lower annualized relapse rates, worsening disability, and worsening MS symptoms following menopause although findings have been variable (20–26). However, in a prior scoping review (2) there were limited or no evaluations of MRI changes (27, 28), menopause symptoms, burdens of MS treatment, co-management of MS and menopausal symptoms, and others. The top-rated questions in the area of menopause aligned with these gaps and focused on response to DMT and quality of life during menopause, effects of MS on symptoms and severity of menopause, and evaluation of whether medications used in MS alter ovarian function or symptoms of menopause. There was also overlap in the menopause-related questions identified by our focus groups with key themes of interest around menopause expressed by a separate online cohort of pwMS (29). There is clear interest and substantial opportunity for further study in this topic.

Our work has several limitations with respect to methodology. First, only those who had access and the capability to use electronic, internet connected devices were able to participate in the global surveys and focus groups. This may exclude individuals with lower socioeconomic status or those with severe disability. However, the NARCOMS registry participants have the option to complete surveys on paper and the findings from the global and NARCOMS surveys were similar. Also, we only offered participation in English, Spanish, and French. Then, we deviated from standard JLA methodology by using electronic platforms and holding focus groups rather than a workshop. This eliminated discussion around prioritization and resulted in different participants engaging with different steps. However, other investigators have modified JLA methodology to include electronic platforms with similar goals of being globally inclusive, and facilitating participant engagement by reducing the need for travel (30). Last, in the NARCOMS registry, the question regarding gender uses terms that reflect sex (female, male) reflecting recommendations for that question at the time it was instituted. We used similar language in the first global survey for comparability between the two surveys but modified options for gender to be appropriate (woman, man) in the final global survey. Future studies should capture sex assigned at birth and gender using appropriate language.

This work has other limitation as well. Despite our efforts to reach a broad audience in our initial by working with multiple organizations and distributing surveys in multiple languages, most respondents were from North America or Europe with little representation from Central America, South America and Africa. In the NARCOMS survey and focus groups, where information on ancestral background was collected, most individuals identified as White, European, and/or Scandinavian with a minority identifying as Black or African; representation of other backgrounds was limited. Respondents and participants may have also had higher levels of educational attainment than the average MS population. Other JLA priority setting efforts have similarly been concerned about the limited reach of underrepresented populations. Also, the average age of respondents and participants for all steps was relatively old. However, this was consistent with the peak age-specific prevalence of MS in the US (31) and, when weighting topic ranking evaluations by age, three of the five high priority topics remained the same. Then, among the respondents who were clinicians and researchers, most were females. It is uncertain whether this reflects differential interest in the topic, gender differences in response rates, or an imbalance in the characteristics of the people surveyed. We suggest that the priorities identified by our study be applied with these limitations in mind. Finally, we focused on women’s health issues in this study, but men’s health also warrants attention and should be the subject of future priority-setting work.

## 5 Conclusion

We hope that this large-scale, modified JLA method priority-setting work will help direct research efforts focused on women’s health issues in MS. Clinicians may also consider including these identified high priority topics and questions during routine clinical discussions. With the elucidation of these priorities, future work is needed to monitor alignment and progress. Additionally, further expansion of this priority setting among groups under-represented in our efforts would be welcomed.

## Data availability statement

The datasets presented in this article are not readily available because of ethical and privacy restrictions. Requests to access the datasets should be directed to the corresponding author.

## Ethics statement

The studies involving humans were approved by the Institutional Review Boards of the Cleveland Clinic and the University of Texas Southwestern. The studies were conducted in accordance with the local legislation and institutional requirements. The ethics committee/institutional review board waived the requirement of written informed consent for participation from the participants or the participants’ legal guardians/next of kin because the consent statement included in the surveys indicated that completion of the anonymous survey implied consent. Participation in focus groups also implied consent as these only generated research priorities.

## On behalf of the International Advisory Committee on Clinical Trials in MS

Past and present members of the International Advisory Committee on Clinical Trials in MS include Maria Pia Amato (University of Florence), Lilyana Amezcua (University of Southern California), Brenda Banwell (University of Pennsylvania), Frederik Barkhof (VU University Amsterdam), Amit Bar-Or (University of Pennsylvania), Helmut Butzkueven (Monash University), Peter Calabresi (Johns Hopkins University), Jeremy Chataway (University College London), Tanuja Chitnis (Brigham and Women’s Hospital), Timothy Coetzee (National MS Society), Jorge Correale (Raul Carrea Institute for Neurological Research), Tobias Derfuss (University Basel), Marcia Finlayson (Queen’s University), Kazuo Fujihara (Fukushima Medical School of Medicine), Myla Goldman (Virginia Commonwealth University), Kerstin Hellwig (Ruhr University), Joep Killestein (MS Center Amsterdam), Dorlan Kimbrough (Duke University), Daphne Kos (University of Leuven), Christine Lebrun-Frenay (Universite Cote d’Azur), Ruth Ann Marrie (University of Manitoba), Aaron Miller (Mount Sinai School of Medicine), Xavier Montalban (Vall d’Hebron University Hospital), Ellen Mowry (Johns Hopkins University), Daniel Ontaneda (Clleveland Clinic), Jiwon Oh (St. Michael’s Hospital), Sudarshini Ramanathan (University of Sydney), Daniel Reich (NIH-National Institute of Neurological Disorders and Stroke), Amber Salter (University of Texas Southwestern), Mara Rocca (Scientific Institute Ospedale San Raffaele), Maria Pia Sormani (University of Genoa), Alan J. Thompson (University College London), Mar Tintore (Vall d’Hebron University Hospital), Helen Tremlett (University of British Columbia), Anneke van der Walt (Monash University), Sandra Vukusic (Hopital Neurologique Pierre Werthemier—GHE), Emmanuelle Waubant (University of California San Francisco), Paola Zaratin (Italian MS Society and Foundation).

## Author contributions

LR: Conceptualization, Formal analysis, Methodology, Project administration, Supervision, Writing – original draft, Resources, Writing – review & editing. MF: Conceptualization, Methodology, Writing – review & editing, Investigation. MA: Conceptualization, Writing – review & editing. JC: Conceptualization, Writing – review & editing. KH: Conceptualization, Writing – review & editing. MT: Conceptualization, Writing – review & editing, Funding acquisition. SV: Conceptualization, Writing – review & editing. AS: Conceptualization, Data curation, Investigation, Methodology, Resources, Writing – review & editing. RM: Conceptualization, Funding acquisition, Methodology, Formal analysis, Project administration, Supervision, Writing – original draft.
