# Supplementary material for: Priority setting: women’s health topics in multiple sclerosis
Source: Front Neurol. 2024 Feb 19;15:1355817. doi: 10.3389/fneur.2024.1355817 (PMC10910071; doi:10.3389/fneur.2024.1355817)
Supplement: Supplementary file 1 [file Data_Sheet_1.docx]

Supplementary Material: Results

Table e1. Characteristics of non-responders and responders to NARCOMs survey

| **Characteristic** | **Non-Responder (N=3499)** | **Responder (N=5098)** | **p-value** |
| --- | --- | --- | --- |
| Females, n (%) | 2797 (80.2) | 4132 (81.1) | 0.39 |
| Race, n (%) |  |  | <0.001 |
| White | 2922 (84.6) | 4455 (87.7) |  |
| Black | 127 (3.7) | 115 (2.3) |  |
| Other | 405 (11.7) | 507 (10.0) |  |
| Education Level, n (%) |  |  | <0.001 |
| High school/GED | 813 (29.6) | 1202 (26.1) |  |
| Associate’s Degree | 434 (15.8) | 668 (14.5) |  |
| Bachelor's Degree | 787 (28.7) | 1399 (30.4) |  |
| Post Bachelor’s Degree | 620 (22.6) | 1222 (26.6) |  |
| Technical Degree | 89 (3.2) | 108 (2.3) |  |
| Age at Symptom Onset (years), mean (SD) | 31.1 (10.4) | 31.4 (.9) | 0.24^a^ |
| Age at Enrollment (years), mean (SD) | 47.8 (10.7) | 47.3 (9.5) | <0.019^a^ |
| PDDS at enrollment, n (%) |  |  | <0.001^c^ |
| Mild (0-1) | 984 (34.8) | 1806 (40.4) |  |
| Moderate (2-4) | 1200 (42.5) | 1855 (41.5) |  |
| Severe (5-8) | 641 (22.7) | 814 (18.2) |  |

*Data not available for all participants. Missing values: age = 58, Sex = 13, Race = 66, Education Level = 1255, Age at Symptom Onset = 901, Age at Enrollment = 827, PDDS Score at Enrollment = 1297. p-values: a=ANOVA, b=Kruskal-Wallis test, c=Pearson's chi-square test, d=Fisher's Exact test.

Table e2. Factors associated with priority rankings in follow-up ANCOVAS: Global survey

| **Priority** | **Type of participant**  **F-value (df), p-value** | **Region**  **F-value (df), p-value** | **Gender**  **F-value (df), p-value** | **Age**  **F-value (df), p-value** |
| --- | --- | --- | --- | --- |
| **Menopause** | 0.78 (2), 0.46 | 4.99 (1), 0.026 | **5.81 (2), 0.0031** | **35.0 (1), <0.0001** |
| **Sexual Dysfunction** | **5.33 (2), 0.005** | **10.8 (1), 0.0011** | 1.21 (2), 0.30 | **9.3 (1), 0.0024** |
| **Pregnancy** | **27.1 (2), <0.0001** | 2.97 (1), 0.085 | 3**.02 (2), 0.049** | **56.8 (1), <0.0001** |
| **Cancer** | **6.63 (2), 0.0014** | 1.34 (1), 0.25 | 2.09 (2), 0.12 | **20.7 (1), <0.0001** |
| **Hormones** | **4.01 (2), 0.018** | **14.8 (1), 0.0001** | 0.17 (2), 0.84 | **4.25 (1), 0.039** |
| **Parenthood** | **6.4 (2), 0.0018** | 1.39 (1), 0.24 | 0.99 (2), 0.37 | **10.5 (1), 0.0012** |
| **Menstrual** | **3.41 (2), 0.033** | 3.52 (1), 0.061 | 0.65 (2), 0.52 | **48.7 (1), <0.0001** |
| **Puberty** | 1.83 (2), 0.16 | 1.14 (1), 0.29 | 0.55 (2), 0.58 | 0.45 (1), 0.50 |
| **Breastfeeding** | **37.1 (2), <0.0001** | 1.09 (1), 0.30 | **3.05 (2), 0.048** | **31.1 (1), <0.0001** |
| **Neonatal** | **5.08 (2), 0.0063** | **3.28 (2), 0.0063** | 0.75 (2), 0.47 | **33.6 (1), <0.0001** |
| **Birth Control** | 0.93 (2), 0.40 | 2.02 (1), 0.16 | 0.75 (2), 0.47 | **21.7 (1), <0.0001** |
| **Fertility** | 1.66 (2), 0.19 | 0.02 (1), 0.90 | 0.61 (2), 0.54 | **40.0 (1), <0.0001** |
| **Domestic violence** | 0.26 (2), 0.77 | 0.77 (1), 0.38 | **3.04 (2), 0.048** | 0.07 (1), 0.79 |
| **Family Planning** | **12.4 (2), <0.0001** | 0.41 (1), 0.52 | 2.8 (2), 0.06 | **4.73 (1), 0.030** |
| **In vitro fertilization** | **19.96 (2), <0.0001** | 0.0 (1), 0.97 | 0.89 (2), 0.41 | **7.31 (1), 0.0069** |
| **Sexually transmitted disease** | 0.77 (2), 0.46 | 0.03 (1), 0.85 | 0.10 (2), 0.90 | 0.0 (1), 0.99 |
| **Gender identity** | 1.54 (2), 0.21 | 0.41 (1), 0.52 | **9.28 (2), <0.0001** | 0.29 (1), 0.59 |
| **Sexual Orientation** | 0.30 (2), 0.74 | 0.02 (1), 0.89 | **12.6 (2), <0.0001** | 0.16 (1), 0.69 |

Type of participant = person with MS, caregiver, other. Region = North American vs. Other

Table e3. Factors associated with priority rankings in follow-up ANCOVAS: Global survey, limited to female respondents with MS

| **Priority** | **Region**  **F-value (df), p-value** | **Direction of Effect** | **Age**  **F-value (df), p-value** | **Direction of Effect** |
| --- | --- | --- | --- | --- |
| **Menopause** | **4.31 (1), 0.038** | NA higher | **36.9 (1), <0.0001** | Higher |
| **Sexual Dysfunction** | **8.73 (1), 0.0032** | NA higher | **11.1 (1), 0.009** | Higher |
| **Pregnancy** | 0.09 (1), 0.76 |  | **65.6 (1), <0.0001** | Lower |
| **Cancer** | 0.82 (1), 0.37 |  | **29.3 (1), <0.0001** | Higher |
| **Hormones** | **9.46 (1), 0.0021** | NA higher | 3.30 (1), 0.070 |  |
| **Parenthood** | 0.41 (1), 0.52 | NA higher | **11.33 (1), 0.0008** | Lower |
| **Menstrual** | 3.75 (1), 0.053 |  | **53.1 (1), <0.0001** | Lower |
| **Puberty** | 1.58 (1), 0.21 |  | 0.93 (1), 0.33 |  |
| **Breastfeeding** | 0.11 (1), 0.74 |  | **29.5 (1), <0.0001** | Lower |
| **Neonatal** | 1.49 (1), 0.22 |  | **41.2 (1), <0.0001** | Lower |
| **Birth Control** | 1.92 (1), 0.17 |  | **23.6 (1), <0.0001** | Lower |
| **Fertility** | **4.86 (1), 0.028** | NA higher | **54.2 (1), <0.0001** | Lower |
| **Domestic violence** | **5.25 (1), 0.022** | NA lower | 0.08 (1), 0.77 |  |
| **Family Planning** | 1.34 (1), 0.25 |  | **4.07 (1), 0.044** | Lower |
| **In vitro fertilization** | 3.02 (1), 0.083 |  | **17.8 (1), <0.0001** | Lower |
| **Sexually transmitted disease** | 0.29 (1), 0.59 |  | 0.02 (1), 0.89 |  |
| **Gender identity** | 0.20 (1), 0.66 |  | 0.11 (1), 0.74 |  |
| **Sexual Orientation** | 0.31 (1), 0.58 |  | 0.08 (1), 0.77 |  |

NA = North America

Table e4. Respondents of the second global survey

| **Characteristic** | **All**  **N = 712** | **Person with MS**  **N = 398** | **Care partner**  **N = 29** | **Other**  **N = 233** |
| --- | --- | --- | --- | --- |
| Age (yrs)*, mean (SD) | 47.5 (13.1) | 46.3 (12.5) | 47.8 (14.1) | 49.3 (13.7) |
| Preferred language, n (%) |  |  |  |  |
| English | 393 (55.2) | 96 (47.3) | 21 (72.4) | 191 (82.0) |
| French | 258 (36.2) | 99 (48.8) | 5 (17.2) | 6 (2.6) |
| Spanish | 61 (8.6) | 8 (3.9) | 3 (10.3) | 36 (15.4) |
| Gender^a^, n (%) |  |  |  |  |
| Women | 574 (86.3) | 200 (98.5) | 19 (67.9) | 154 (66.3) |
| Men | 89 (13.4) | 3 (1.5) | 9 (32.1) | 76 (32.8) |
| Neither man nor woman/ Prefer not to say | 2 (0.3) | 0 (0) | 0 (0) | 2 (0.9) |
| Type of respondent^b^, n (%) |  |  |  |  |
| Person with MS | 398 (60.3) |  |  |  |
| Care partner | 29 (4.4) |  |  |  |
| Not a person with MS or care partner | 233 (35.3) |  |  |  |
| Type of MS^c^, n (%) |  |  |  |  |
| Clinically isolated syndrome |  | 5 (2.5) |  |  |
| Relapsing remitting |  | 133 (66.2) |  |  |
| Secondary progressive |  | 32 (15.9) |  |  |
| Primary progressive |  | 23 (11.4) |  |  |
| Don’t know/unsure |  | 8 (4.0) |  |  |
| Work Sector^d^, n (%) |  |  |  |  |
| Government |  |  |  | 15 (6.4) |
| Health care/Health professional |  |  |  | 167 (71.7) |
| Non-profit |  |  |  | 19 (8.2) |
| Research in academic/health care setting |  |  |  | 79 (33.9) |
| Research in commercial/industry setting |  |  |  | 10 (4.3) |
| Industry setting, non-research |  |  |  | 3 (1.3) |
| Other |  |  |  | 5 (2.2) |
| Organizational role^d^, n (%) |  |  |  |  |
| Administrative |  |  |  | 16 (6.9) |
| Clinical care |  |  |  | 162 (69.5) |
| Patient advocacy |  |  |  | 9 (3.9) |
| Research and evaluation |  |  |  | 122 (52.4) |
| Other |  |  |  | 17 (7.3) |
| Professional background^d^, n (%) |  |  |  |  |
| Administrator |  |  |  | 6 (2.6) |
| Physician |  |  |  | 143 (61.4) |
| Nurse |  |  |  | 19 (8.2) |
| Physiotherapist |  |  |  | 5 (2.2) |
| Occupational therapist |  |  |  | 4 (1.7) |
| Social worker |  |  |  | 0 (0) |
| Speech therapist |  |  |  | 1 (0.4) |
| Researcher – clinical |  |  |  | 70 (30.0) |
| Research – health systems and services |  |  |  | 6 (2.6) |
| Researcher – biomedical |  |  |  | 27 (11.6) |
| Researcher – population hlth/epi |  |  |  | 16 (6.9) |
| Other |  |  |  | 27 (11.6) |
| Affiliation |  |  |  |  |
| TRIMS** | 179 (25.1) |  |  |  |
| AAN | 80 (11.2) |  |  |  |
| Chinese Neurol. Assoc | 0 (0) |  |  |  |
| CMSC | 74 (10.4) |  |  |  |
| EAN | 35 (4.9) |  |  |  |
| ECF | 14 (2.0) |  |  |  |
| EFNS | 7 (1.0) |  |  |  |
| EMSP | 28 (3.9) |  |  |  |
| GNS | 4 (0.6) |  |  |  |
| iWiMS | 23 (3.2) |  |  |  |
| MSIF | 43 (6.0) |  |  |  |
| MS Societies | 189 (26.5) |  |  |  |
| RIMS | 16 (2.3) |  |  |  |
| Other | 79 (11.1) |  |  |  |
| None | 243 (34.1) |  |  |  |

**b- n = 52 missing; c – n = 2 missing**

**TRIMS include Americas Committee for Treatment and Research in MS (ACTRIMS), Brazilian Committee for Treatment and Research in MS (BCTRIMS), European Committee for Treatment and Research in MS (ECTRIMS), Latin American Committee for Treatment and Research in MS (LACTRIMS), Middle East North Africa Committee for treatment and Research in MS (MENACTRIMS), Committee for treatment and Research in MS MEXTRIMS, Pan-Asian Committee for treatment and Research in MS (PACTRIMS), Russian Committee for treatment and Research in MS (RUCTRIMS), Sri Lanka Committee for treatment and Research in MS (SLTRIMS). Note some individuals reported these affiliations even if the survey was not directly distributed to those organizations.

AAN = American Academy of Neurology, CMSC = Consortium of MS Centers, EAN = European Academy of Neurology, ECF = European Charcot Foundation, EFNS = , EMSP =, GNS = German Neurological Society, iWiMS = International Women in Multiple Sclerosis, MS = multiple sclerosis, RIMS = Rehabilitation in MS

**Figure e1. Flowchart illustrating study components and participants at each stage**

**
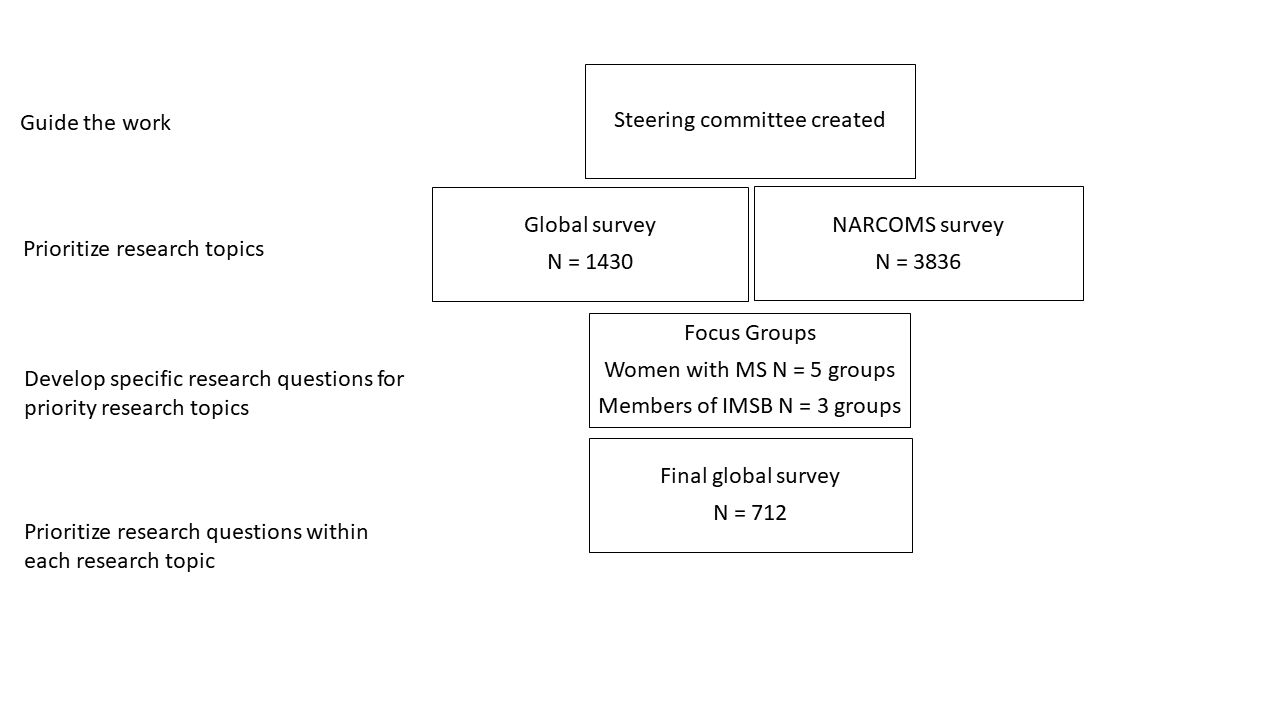
**

**Figure e2.** Geographic distribution of respondents to final global survey. Inner ring = persons with multiple sclerosis, middle ring = care partners, outer ring = all others
