# Supplementary material for: Priority setting: women’s health topics in multiple sclerosis
Source: Front Neurol. 2024 Feb 19;15:1355817. doi: 10.3389/fneur.2024.1355817 (PMC10910071; doi:10.3389/fneur.2024.1355817)
Supplement: Supplementary file 2 [file Data_Sheet_2.docx]

Supplementary Material: Appendix I-III

Appendix I. Global Survey

Thank you for taking the time to participate in this brief survey about research priorities regarding women’s health in multiple sclerosis (MS). This survey is designed to determine what are deemed to be the most important research priorities in this area for women living with MS.

About you

1. What is your gender?
2. Female
3. Male
4. Neither male nor female
5. What is your age in years? ________
6. What country do you live in? _______
7. Are you a person living with MS or a carepartner for someone with MS?
8. Yes, I have MS
9. Yes, I am a caregiver for someone with MS (if yes, skip logic to Q10)
10. No (if no, skip logic to Question 6)
11. What type of MS has your doctor said you have now? **Select one**

| 1. **Clinically Isolated Syndrome (CIS)**:   Had only one attack ever, without further attacks or new MRI activity since diagnosis |
| --- |
| 1. **Relapsing Remitting:** Periods of worsening with all or some recovery |
| 1. Secondary Progressive: Started with relapsing remitting MS, but then developed gradually increasing disability, even between relapses |
| 1. **Primary Progressive:** Increasing disability from onset without relapses |
| 1. **Don’t know/Unsure** |

- Skip to Question 9

1. What sector do you work in? Please select all that apply.
2. Government
3. Health Care/ Health Professional
4. Non-Profit Organization
5. Research in academic or health care setting
6. Research in commercial/industry setting
7. Industry setting, non-research
8. Other (please specify)_________________________
9. What is your role in your organization? Please select all that apply.
10. Administrative
11. Clinical Care
12. Patient Advocacy
13. Research and Evaluation
14. Other (please specify)
15. What is your professional background? Please select all that apply.
16. Administrator
17. Physician
18. Nurse
19. Physiotherapist
20. Occupational therapist
21. Social worker
22. Speech therapist
23. Researcher – Clinical
24. Researcher – Health systems and services
25. Researcher – Biomedical
26. Researcher – Population health or epidemiology (social, cultural, environmental factors)
27. Other, please specify:
28. What organizations are you affiliated with? (Mark all that apply)
29. ACTRIMS
30. American Academy of Neurology
31. BCTRIMS
32. Consortium of MS Centers
33. ECTRIMS
34. European Academy of Neurology (EAN)
35. European Federation of Neurological Societies (EFNS)
36. European Multiple Sclerosis Platform (EMSP)
37. iWiMS
38. LACTRIMS
39. PACTRIMS
40. MENACTRIMS
41. Multiple Sclerosis International Federation (MSIF)
42. National or local Multiple Sclerosis Societ(ies)
43. Rehabilitation in Multiple Sclerosis (RIMS)
44. RUCTRIMS
45. German Neurological Society
46. Other (specify______________________)
47. None

**Women’s Health in MS: Research Area**

1. With respect to the issue of **women’s health** in MS, what are the *most important* research areas in general? For example, if you thought one of the most important research questions was whether menopause affects symptoms of MS you would rank menopause as important. If you thought one of the most important questions was whether it is safe to take disease-modifying therapies while breastfeeding you would rank breastfeeding as important. Please rank your top 5 choices in order of importance (#1 being the most important).
2. Puberty (e.g. effects on symptoms in pediatric-onset MS)
3. Menstrual cycle (e.g. perimenstrual symptom worsening)
4. Contraception/Birth control
5. Pregnancy (incl. pregnancy loss)
6. Neonatal outcomes/childhood development
7. Fertility/Infertility
8. Assisted reproduction (e.g., in vitro fertilization)
9. Breastfeeding
10. Menopause and hormone replacement therapy
11. Sex hormones and influence on MS outcomes or as treatment
12. Sexual dysfunction
13. Parenthood (effects of MS on being a parent)
14. Family planning services (e.g. pregnancy and abortion counseling, pregnancy testing)
15. Gynecologic Cancer and cancer screening (e.g. Breast, Cervical, Ovarian)
16. Sexually transmitted diseases
17. Sexual orientation (e.g. heterosexual, lesbian)
18. Gender Identity (e.g. transgender, queer)
19. Intimate partner violence
20. Are there other general **topics** that should be considered that were not listed in the previous question?
    1. No
    2. Yes (specify _________________________________________________________)
21. Would you be willing to participate in another survey or in a focus group regarding this topic? If you answer yes, you will be taken to a separate survey site to enter your name and contact information.
    1. Yes, in a survey
    2. Yes, in a focus group
    3. Yes, in a survey and focus group
    4. No

Thank you for your participation. It is much appreciated.

Appendix II. Response options for NARCOMS Survey

We categorized race and ethnicity as White, Black/African American and Other. Annual household income was categorized as <$50,000, $50,001-$100,000, >$100,000 and “I do not wish to answer”. Participants reported if a doctor had diagnosed them with any of the following comorbidities: anxiety disorder, depression, autoimmune thyroid disease, diabetes, hypertension, hyperlipidemia, heart disease including myocardial infarction, chronic lung disease, irritable bowel syndrome, psoriasis, fibromyalgia, sleep apnea, migraine, stroke, kidney disease and cancer. We combined all of these comorbidities except depression and anxiety into a count (0, 1, 2, ≥3) of ‘physical’ comorbidities. We described health behaviors as current smoking (yes/no), any physical activity (yes/no), alcohol intake (any vs none). We assessed disability status using Patient Determined Disease Steps (PDSS), which includes one question with eight possible responses ranging from 0 (normal) to 8 (bedridden). The self-reported PDDS is strongly correlated with a clinician-assessed Expanded Disability Status Scale Score (r = 0.73).[^6^](#_ENREF_6) Consistent with prior work, we classified the PDDS as mild (0-1), moderate (2-4) and severe (5-8).[^7^](#_ENREF_7)

Appendix III. Focus Group Participant Survey

Hello!

This survey is being conducted on behalf of the International Advisory Committee on Clinical Trials in MS as part of the focus group study to identify important research questions on women’s health issues in MS. Please complete this survey ahead of your focus group session.

Please hit the “Submit” button when you have completed the survey.

Thank you for participating!

1. What is your age in years? ______
2. Stakeholder group to which the focus group participant belongs (check all the apply)
   1. Person with MS
   2. Care partner of person with MS (skip to question 4)
   3. Clinician (skip to question 4)
   4. Research (skip to question 4)
   5. Patient advocate (skip to question 4)
   6. Other (skip to question 4)
3. Current MS Disease Course

| 1. **Clinically Isolated Syndrome (CIS)**: Had only one attack ever, without further attacks or new MRI activity since diagnosis |
| --- |
| 1. **Relapsing Remitting:** Periods of worsening with all or some recovery |
| 1. Secondary Progressive: Started with relapsing remitting MS, but then developed gradually increasing disability, even between relapses |
| 1. **Primary Progressive:** Increasing disability from onset without relapses |
| 1. **Don’t know/Unsure** |

1. What country do you live in? ___________
2. What is your gender?
   1. Man
   2. Woman
   3. Transgender Man
   4. Transgender Woman
   5. Non-binary
   6. Prefer not to say
3. People around the world come from many different cultural and racial backgrounds. Please check all that apply
   1. Scandinavian/ White (Norway/ Sweden)
   2. Northwest European/ White (UK, France, Germany, ect.)
   3. Eastern European/ White (Poland, Hungary, ect.)
   4. Southern European/ White (Greece, Italy, ect.)
   5. Chinese
   6. South Asian (East Indian, Pakistani, Sri Lankan, ect.)
   7. African/ Black
   8. Filipino
   9. Latin American (Mexico, Latin Caribbean, Central and South American)
   10. Southeast Asian (Cambodian, Indonesia, Laotian, Vietnamese, ect.)
   11. Arab
   12. West Asian (Afghan, Iranian, ect.)
   13. Japanese
   14. Korean
   15. Indigenous Peoples (Native American, First Nations, Pacific Islander, ect.)
   16. Other
   17. Refuse to Answer
4. Desire to receive results of study once completed? Yes/no
   1. If yes, Enter email to receive results ________
